# Study of Early Onset Schizophrenia: Associations of *GRIN2A* and *GRIN2B* Polymorphisms

**DOI:** 10.3390/life11100997

**Published:** 2021-09-22

**Authors:** Evgeniya G. Poltavskaya, Olga Yu. Fedorenko, Elena G. Kornetova, Anton J. M. Loonen, Alexander N. Kornetov, Nikolay A. Bokhan, Svetlana A. Ivanova

**Affiliations:** 1 Mental Health Research Institute, Tomsk National Research Medical Center of the Russian Academy of Sciences, 634014 Tomsk, Russia; f_o_y@mail.ru (O.Y.F.); kornetova@sibmail.com (E.G.K.); bna909@gmail.com (N.A.B.); ivanovaniipz@gmail.com (S.A.I.); 2 Fundamental Psychology and Behavioral Medicine Department, Siberian State Medical University, 634050 Tomsk, Russia; alkornetov@gmail.com; 3 Unit of PharmacoTherapy, Epidemiology & Economics, Groningen Research Institute of Pharmacy, University of Groningen, 9713AV Groningen, The Netherlands; a.j.m.loonen@rug.nl

**Keywords:** schizophrenia, genetic polymorphism, *GRIN2A*, *GRIN2B*, genes, early onset

## Abstract

Background: Schizophrenia is a complex mental disorder with a high heritability. Dysfunction of the N-methyl-D-aspartate (NMDA)-type glutamate receptors may be involved in the pathogenesis of schizophrenia. In this study, we examined the contribution of *GRIN2A* and *GRIN2B* (Glutamate Ionotropic Receptor NMDA Type Subunit 2A/2B) polymorphisms to the clinical features of schizophrenia, such as the leading symptoms, the type of course, and the age of onset. Methods: A population of 402 Russian patients with schizophrenia from the Siberian region was investigated. Genotyping of seventeen single-nucleotide polymorphisms (SNPs) in *GRIN2A* and *GRIN2B* was performed using QuantStudio™ 3D Digital PCR System Life Technologies amplifier using TaqMan Validated SNP Genotyping Assay kits (Applied Biosystems). The results were analyzed using Chi-square and the Fisher’s exact tests. Results: We found an association of *GRIN2A* rs7206256 and rs11644461 and *GRIN2B* rs7313149 with the early onset (before the age of 18 years old) schizophrenia. We did not reveal any associations of *GRIN2A* and *GRIN2B* polymorphisms with leading (positive vs. negative) symptoms or type of course (continuous vs. episodic) of schizophrenia. Conclusions: In the study, we confirmed the involvement of the *GRIN2A* and *GRIN2B* genes in the early onset of schizophrenia in a Russian population of the Siberian region.

## 1. Introduction

Schizophrenia is a complex mental disorder that seems to originate from disruption of brain development and/or maturation caused by genetic or environmental factors, or both [1]. The family, twin and adoption studies have demonstrated a high heritability of the disease which is not simply defined by several major genes but rather evolves from addition or potentiation of a specific cluster of genes, which subsequently determines the genetic vulnerability of an individual [2].

The oldest and best developed idea for the pathophysiology of schizophrenia is the thought that this disorder is caused by a dysfunction within the dopaminergic system [3]. Now, it is almost impossible to imagine that such a dysfunction would be independent of the dysfunction of the gamma aminobutyric acid (GABA) and glutamate neurotransmitter systems [4]. Involvement of glutamatergic neurotransmission is therefore at the heart of another main hypothesis [5,6]. Glutamate is the most important excitatory neurotransmitter of the central nervous system and affects the transmission of impulses via ionotropic and metabotropic receptors [7]. Ionotropic N-methyl-D-aspartate (NMDA) type glutamate receptors are of particular interest in this context because they increase the sensitivity of certain synapses through long-term potentiation (LTP) and lead to excitotoxicity when stimulated too massively.

Dysfunction of the N-methyl-D-aspartate (NMDA)-type glutamate receptor has been proposed as a mechanism in the etiology of schizophrenia, based on the observation that non-competitive antagonists of the NMDA receptor, such as phencyclidine, induce schizophrenia-like symptoms [5,6]. Psychiatric diseases may occur when genes which encode receptors become dysfunctional [8]. Dysfunction of NMDA receptors may therefore in principle be associated with the occurrence of neurodevelopmental changes that may underlie the pathology of schizophrenia [1]. In addition, neuronal excitotoxicity resulting from hyperactive NMDA receptors is suggested to play a role in many brain disorders, including Alzheimer’s disease and schizophrenia [9,10]. At the same time, in humans, depending on dose, blocking the NMDA receptor with ketamine can cause antidepressant effects [11].

The NMDA receptors are ionotropic glutamate receptors, composed of a heterotetramer of two NMDA 1 (GRIN1) subunits and archetypally two GRIN2 (or GluN2) subunits of which four variants (GRIN2A-GRIN2D) exist, combined in a varying ratio to make up the receptor complex [12]. GluN2A, encoded by the *GRIN2A* gene, is the most abundant of the GluN2 NMDA receptor subunits in the mammalian CNS. Physiological and genetic evidence implicate GluN2A-containing receptors in susceptibility to autism, schizophrenia, and childhood epilepsy and neurodevelopmental disorders such as Rett Syndrome [13]. A previous study identified a variable (GT)n polymorphism in the promoter region of the GluN2A subunit gene (*GRIN2A*), and showed its association with schizophrenia in a case–control study, together with a correlation between the length of the repeat and the severity of chronic outcome [14].

The *GRIN2B* (glutamate receptor, ionotropic, N-methyl-D-aspartate 2B) gene, located in the short arm of chromosome 12, encoding the NR2B subunit of the N-methyl-D-aspartate receptor, has recently been recognized to play an important role in corticogenesis and brain plasticity. Deletions in the short arm of chromosome 12 are rare. Hemizygous loss of function of the *GRIN2B* gene results in developmental delay, whereas gain of function leads to epilepsy, and infantile spasms in particular. In addition, *GRIN2B* variants have been associated with autism spectrum disorder and schizophrenia [15]. It has also been demonstrated that the level of an ionotropic N-methyl-D-aspartate 2B subunit (*GRIN2B*) of the glutamate receptor tends to increase after subchronic administration of clozapine, suggesting that *GRIN2B* may play an active role in the pathogenesis of schizophrenia and the function of clozapine medication [16].

Earlier, we studied the role of *GRIN2A* and *GRIN2B* variants in antipsychotic-induced tardive dyskinesia in schizophrenia in terms of genetically determined increase in vulnerability to NMDA-induced excitotoxicity [17,18,19].

Schizophrenia is highly heterogeneous mental disorder. The course of the disease can be either continuous or episodic, which significantly affects treatment management. Some of the clinical characteristics of the disease act as a poor prognostic sign. Stable negative symptoms are associated with a higher progression of schizophrenia. Also, it is generally accepted that childhood-onset schizophrenia is less benign compared to a debut after puberty. In the long term, many patients with childhood-onset are expected to have poor social adaptability and severe functional impairments [20,21].

In this study, we examined the contribution of *GRIN2A* and *GRIN2B* polymorphisms to the clinical features of schizophrenia, specifically the leading symptoms (negative or positive), course type (continuous or episodic), as well as the age of onset of the disease.

## 2. Materials and Methods

### 2.1. Patients

This study was carried out in accordance with The Code of Ethics of the World Medical Association (Declaration of Helsinki 1975, revised in Fortaleza, Brazil, 2013) for experiments involving humans and according to the protocol approved by the Bioethical Committee of the Mental Health Research Institute of the Tomsk National Research Medical Center of the Russian Academy of Sciences (protocol N63 of 14.07.2014). We recruited 402 patients with schizophrenia being treated at clinics of the Mental Health Research Institute, Siberian State Medical University, Tomsk Clinical Psychiatric Hospital (Tomsk), Kemerovo Regional Clinical Psychiatric Hospital (Kemerovo) in the Siberian region. The inclusion criteria were a verified clinical diagnosis of schizophrenia (F20) according to the World Health Organization World Mental Health Composite International Diagnostic Interview (WHO WMH-CIDI) for schizophrenia diagnostics [22], age 18–60 years, and the patient’s informed consent. Exclusion criteria for all patients were non-Caucasian physical appearance (e.g., Mongoloid, Buryats or Khakassians), organic mental disorders (e.g., epilepsy, Parkinson’s disease) or somatic disorders in the stage of decompensation. Clinical examination and diagnostic assessment were carried out using the Positive and Negative Syndrome Scale (PANSS) [23]. The total PANSS score in the group of patients with schizophrenia was 102 (92; 109) (median and lower-upper quartiles: Me (Q1; Q3)). The course of schizophrenia (continuous or episodic) was determined by ICD-10—in the classification of ICD-10, the fifth character is used for this.

To study the associations between *GRIN2A* and *GRIN2B* polymorphisms and leading symptoms (negative or positive) the total group of 402 patients with schizophrenia was divided into 2 subgroups according to the PANSS survey data: a subgroup of 201 patients with leading negative symptoms and a subgroup of 185 patients with leading positive symptoms. The rest of the patients were not included in the comparison due to mixed symptoms and the lack of prevalence of positive or negative symptoms according to the PANSS. To study the role of *GRIN2A* and *GRIN2B* polymorphisms in the development of the course of schizophrenia, the total group of patients with schizophrenia was divided into 2 subgroups: 297 cases were continuously ill while the remaining cohort of 105 patients experienced an episodic course of the disease. To study the associations between the *GRIN2A* and *GRIN2B* polymorphisms and the age of onset of schizophrenia, a total group of 402 patients with schizophrenia was divided into subgroups: a subgroup of 71 patients with early onset of the disease (before the age of 18 years old) and a subgroup of 331 people with adult-onset schizophrenia (after the age of 18 years old). The group of patients with schizophrenia was divided according to the age of onset of the disease, since the early onset of schizophrenia is interpreted as a risk factor for a more severe course of the disease [24].

### 2.2. Genetic Analysis

Blood samples were taken after 8 h overnight fasting in tubes containing EDTA and stored in several aliquots at −20 °C until DNA isolation using the standard phenol-chloroform method.

Inclusion criteria for SNP selection and genotype are described elsewhere [17]. Genotyping of eleven single-nucleotide polymorphisms (SNPs) in *GRIN2A* (rs7206256, rs1345423, rs8049651, rs9989388, rs7192557, rs9788936, rs9921541, rs11646587, rs1650420, rs11644461, rs4782039) and six SNPs in *GRIN2B* (rs220599, rs7313149, rs2192970, rs10845838, rs10772715, rs1805481) was carried out using a QuantStudio™ 3D Digital PCR System Life Technologies amplifier (Applied Biosystems, Waltham, MA, USA) using TaqMan Validated SNP Genotyping Assay kits (Applied Biosystems) based at The Core Facility “Medical Genomics”, Tomsk National Research Medical Center of the Russian Academy of Sciences.

### 2.3. Statistical Analysis

Statistical analysis was performed using SPSS software for Windows, release 21. The genotype frequencies were checked for Hardy–Weinberg equilibrium using the chi-square test. Chi-square test and the Fisher’s exact test, where necessary, were used for between-group comparisons of genotype or allele frequencies at a significance level of *p* < 0.05. Assessment of the association of genotypes and alleles of the studied polymorphic variants of genes with a pathological phenotype was carried out using the odds ratio (OR) with a 95% confidence interval for the odds ratio (95% CI).

## 3. Results

Details about the studied patient population are presented in Table 1.

The prevalence of schizophrenia is higher among men than among women. This is probably related to the well-known gender differences in the distribution of the incidence and prevalence of schizophrenia [25]. At a relatively young age, as is the case in our population, men are clearly in excess, but at a later age schizophrenia debuts much more frequently in women then in men

Deviation from the HWE was found for *GRIN2A* rs7192557 and GRIN2B rs220599; hence, these polymorphisms were excluded from further consideration.

### 3.1. Association of GRIN2A and GRIN2B Polymorphisms with Leading (Positive vs. Negative) Symptoms of Schizophrenia

The genotypes and alleles frequency analysis of *GRIN2A* and *GRIN2B* polymorphisms detected no “modifier loci” regarding positive or negative leading symptoms of schizophrenia.

### 3.2. Association of GRIN2A and GRIN2B Polymorphisms with Type of Course of Schizophrenia (Continuous Course vs. Episodic Course)

The genotypes and alleles frequency analysis found no difference between *GRIN2A* and *GRIN2B* polymorphisms in patients with schizophrenia with a continuous course of schizophrenia and those with an episodic course of schizophrenia.

### 3.3. Association of GRIN2A and GRIN2B Polymorphisms with Age of Onset of Schizophrenia (Early vs. Adult)

When comparing groups of patients with early age of onset of schizophrenia and adult age of onset of the disease, we found an association of the rs7206256 (*p* = 0.043 for genotypes, *p* = 0.020 for alleles) and rs11644461 (*p* = 0.034 for genotypes, *p* = 0.030 for alleles) polymorphisms of the *GRIN2A* gene (Table 2) and the rs7313149 polymorphism of the *GRIN2B* gene (*p* = 0.048 for alleles) (Table 3) with the early onset of schizophrenia.

Table 4 shows the odds ratio data calculated for polymorphisms associated with age of onset of schizophrenia. In this connection, a group of patients with an early onset of the disease was taken as the main group in the calculation, since an early onset is considered a negative factor in the prognosis of the disease.

In our study, we identified an association of *GRIN2A* rs7206256*A, *GRIN2A* rs1164446*GG/G, and *GRIN2B* rs7313149*A with the early onset of schizophrenia.

## 4. Discussion

N-methyl-D-aspartate receptors mediate a slow calcium-permeable component of excitatory synaptic transmission in the central nervous system. The NMDA receptors are very important for proper brain development and neuroplasticity while hyperfunction results in excitotoxicity associated with the genesis of various neurodegenerative diseases [26]. The *GRIN2A* gene encodes the GluN2A subunit of the NMDA receptor. The GluN2A subunit plays a critical role during postnatal brain development as its expression increases while Glun2B (encoded by the *GRIN2B* gene) expression decreases. Mutations and polymorphisms in the *GRIN2A* gene, coding for GluN2A, are linked to developmental brain disorders such as mental retardation, epilepsy, and mental disorders. This might also apply for schizophrenia. Published data suggest that GluN2A is involved in maturation and phenotypic maintenance of parvalbumin interneurons, and these interneurons suffer from a deficient glutamatergic neurotransmission via GluN2A-containing NMDA receptors in schizophrenia [27].

The *GRIN2B* gene encodes a subunit of the NMDA receptor ion channel, which acts as an agonist binding site for glutamate. The *GRIN2B* gene plays a crucial role in normal neuronal development and is important for learning and memory. Mutations in human *GRIN2B* were distributed throughout the entire gene in a number of patients with various neuropsychiatric and developmental disorders [28].

The *GRIN2A* and *GRIN2B* genes are being actively investigated as candidate genes for the predisposition of schizophrenia and other neurodevelopmental and/or neurodegenerative disorders.

A review by Myers et al. (2019) compares the available information describing the clinical and functional consequences of genetic variations in *GRIN2A* and *GRIN2B*. They showed that *GRIN2A* variants are commonly associated with an epileptic phenotype but that *GRIN2B* variants are commonly found in patients with neurodevelopmental disorders [29]. Krzystanek et al. (2021) did not find any associations between selected nucleotide variants in *GRIN1*, *GRIN2A*, and *GRIN2B* and resistance to clozapine and the presence of cognitive deficits in schizophrenia in a targeted group of 45 Polish patients with super clozapine-resistant schizophrenia and cognitive impairment [30]. We have in the past studied the relationship between *GRIN2A* and *GRIN2B* variants and the occurrence of drug-induced dyskinesia [17,18]. We revealed the susceptibility to levodopa-induced dyskinesia in patients with Parkinson’s disease to be associated with two NMDA receptor (GRIN2A) variants, identified by SNPs rs7192557 and rs8057394, which were previously found to be associated with the age of dyskinesia onset in Huntington’s disease [17]. We hypothesized that this is related to increased vulnerability to neurotoxic damage [19].

The significance of genetic variation of GRIN2A and GRIN2B fits well with the glutamate hypothesis for the pathophysiology of schizophrenia [5,6]. It is hypothesized that positive psychotic symptoms of schizophrenia are possibly mainly attributed to presynaptic dopaminergic changes, whereas negative and cognitive symptoms could be associated with glutamatergic dysfunction. Now, glutamatergic neurotransmission is one of the most widespread neurotransmission systems in the central nervous system, with, for example, virtually all intracortical and corticofugal connections using this neurotransmitter substance [7]. There are therefore many possibilities for circuits and pathways through which glutamatergic neurotransmission can modulate dopaminergic activity [4] and glutamatergic variation need not exclude mediation via dopaminergic neurotransmission. This also brings into focus our hypothesis about the role of the dorsal diencephalic conduction system (DDCS) in the development of mental illnesses such as schizophrenia [31,32,33]. This pathway, via the habenuloid complex between the forebrain and the ascending dopaminergic, adrenergic and serotonergic pathways from the midbrain, is phylogenetically very old and its caudal part has remained largely unchanged since the earliest vertabrates. Within this DDCS, glutamatergic neurotransmission plays a dominant role and the associated neuroplastic changes lead in the initiation and control of life-determining behaviours. It is quite conceivable that *GRIN2A* and *GRIN2B*, via this DDCS, modulate the activity of the ascending monoaminergic pathways and thus cause the ‘positive’ component of schizophrenia [34], while changes in the prefrontal cortex and hippocampus cause negative and cognitive components to arise [5,6]. Whether this involves neurodevelopmental or neurodegenerative changes remains to be seen. Research suggests that cognitive decline does not manifest until puberty/adolescence in schizophrenia patients, although this can be as early as the age of 10 years [35].

Dysfunction of the glutamate receptor N-methyl-D-aspartate is also being studied regarding the particularities of the course of schizophrenia. Thus, previous study has shown a correlation between the duration of the *GRIN2A* variable polymorphism (GT)n repeat and the severity of the chronic outcome of schizophrenia [3]. This may indicate that dysfunction of NMDA receptor subunits can serve as a trigger for the development of schizophrenia, and significant mutations in the *GRIN2A* and *GRIN2B* genes can affect early or late onset. Studies in rat models have shown that *GRIN2A* and *GRIN2B* expression is upregulated in the prefrontal cortex during childhood and adolescence, while a model of schizophrenia is characterized by an immature neurobiological phenotype with reduced *GRIN2A* and *GRIN2B* expression [36]. This suggests the importance of the age at which dysfunction of the NMDA receptor subunits triggers the pathological defect.

In this study, we did not identify associations of the *GRIN2A* and *GRIN2B* genes with leading symptoms or course types of schizophrenia. At the same time, we were the first to obtain data on the association of the *GRIN2A* (rs7206256, rs11644461) and *GRIN2B* (rs7313149) genes with early onset of schizophrenia in the Russian population of the Siberian region.

Earlier onset is often associated with a poorer outcome [37]. Promotion of good mental health, prevention, and early intervention before/at the onset of mental disorders improve outcomes [38]. We could speculate that our findings could be potentially useful for families with psychiatric burden when they pass prenatal genetic counseling for psychiatric disorders, although our data need to be confirmed on a larger sample.

Perhaps, in the future our findings will be significant in terms of treatment of early onset schizophrenia. We did not verify the treatment, such as the pharmacological profile of individual drugs including antipsychotics, since we were not able to track treatment throughout the course of the disease. The lack of verification for treatment may act as a limitation of this study in terms of studying the type of schizophrenia course or leading symptoms and, at the same time, does not play a role in studying the onset of the disease.

Converging evidence implicates redox dysregulation as a pathological mechanism driving the emergence of psychosis. Increased oxidative damage and decreased capacity of intracellular redox modulatory systems are consistent findings in persons with schizophrenia as well as in persons at clinical high risk who subsequently developed frank psychosis. Molecules regulating interneuron function under redox control include NMDA receptor subunits [39].

## 5. Conclusions

Our findings showed no contributions of *GRIN2A* and *GRIN2B* polymorphisms into genetic architecture of schizophrenia’s clinical heterogeneity concerning different leading symptoms (negative or positive) or the course of schizophrenia (continuous or episodic) in our Russian population of the Siberian region. However, we found associations of *GRIN2A* rs7206256 and rs11644461 and *GRIN2B* rs7313149 with the early onset of the disease.

## Figures and Tables

**Table 1:** Demographic and clinical parameters of the studied patients.

**Sample Size, n**	**402**
**Gender, n (%)**	Men: 256 (63.7%) Women: 146 (36.3%)
**Age, years, Me (Q1; Q3)**	41 (29; 53)
**Age at onset, years, Me (Q1; Q3)**	23 (19; 31)
**Duration of illness, years, Me (Q1; Q3).**	14 (7; 25)

**Table 2:** Frequency distribution of genotypes and alleles of *GRIN2A* polymorphisms in patients with schizophrenia with early or adult age of onset of schizophrenia.

SNPs	Genotypes and Alleles	Early Onset (<18 y.o.)	Adult Onset (≥18 y.o.)	χ^2^	*p*-Value
rs7206256	AA	31 (44.9%)	112 (34.1%)	6.279	0.043 *
	AG	34 (39.3%)	162 (49.4%)		
	GG	4 (5.8%)	54 (16.5%)		
	A	96 (69.6%)	386 (58.8%)	5.497	0.020 *
	G	42 (30.4%)	270 (41.2%)		
rs1345423	AA	22 (31.4%)	128 (39.0%)	1.809	0.405
	AC	36 (51.4%)	158 (48.2%)		
	CC	12 (17.1%)	42 (12.8%)		
	A	80 (57.1%)	414 (63.1%)	1.745	0.187
	C	60 (42.9%)	242 (36.9%)		
rs8049651	AA	5 (7.0%)	25 (7.6%)	1.288	0.525
	AG	35 (49.3%)	139 (42.0%)		
	GG	31 (43.7%)	167 (50.5%)		
	A	45 (31.7%)	189 (28.5%)	0.559	0.455
	G	97 (68.3%)	473 (71.5%)		
rs9989388	AA	5 (7.1%)	15 (4.7%)	1.049	0.592
	AG	23 (32.9%)	120 (37.4%)		
	GG	42 (60.0%)	186 (57.9%)		
	A	33 (23.6%)	150 (23.4%)	0.003	0.959
	G	107 (76.4%)	492 (76.6%)		
rs9788936	AA	49 (72.1%)	204 (63.4%)	3.154	0.207
	AG	19 (27.9%)	109 (33.9%)		
	GG	0 (0%)	9 (2.8%)		
	A	117 (86.0%)	517 (80.3%)	2.440	0.119
	G	19 (14.0%)	127 (19.7%)		
rs9921541	AA	4 (5.9%)	13 (4.1%)	1.005	0.605
	AC	21 (30.9%)	115 (36.4%)		
	CC	43 (63.2%)	188 (59.5%)		
	A	29 (21.3%)	141 (22.3%)	0.063	0.802
	C	107 (78.8%)	491 (77.7%)		
rs11646587	AA	5 (7.1%)	21 (6.5%)	0.737	0.692
	AG	26 (37.1%)	138 (42.7%)		
	GG	39 (55.7%)	164 (50.8%)		
	A	36 (25.7%)	180 (27.9%)	0.267	0.606
	G	104 (74.3%)	466 (72.1%)		
rs1650420	AA	8 (11.3%)	56 (17.2%)	2.788	0.248
	AG	38 (53.5%)	142 (43.6%)		
	GG	25 (35.2%)	128 (39.3%)		
	A	54 (38.0%)	254 (39.0%)	0.042	0.837
	G	88 (62.0%)	398 (61.0%)		
rs11644461	AA	25 (35.2%)	143 (43.2%)	6.760	0.034 *
	AG	30 (42.3%)	151 (45.6%)		
	GG	16 (22.5%)	37 (11.2%)		
	A	80 (56.3%)	437 (66.0%)	4.767	0.030 *
	G	62 (43.7%)	225 (34.0%)		
rs4782039	AA	38 (53.5%)	190 (58.1%)	0.915	0.633
	AG	31 (43.7%)	124 (37.9%)		
	GG	2 (2.8%)	13 (4.0%)		
	A	107 (75.4%)	504 (77.1%)	0.192	0.662
	G	35 (24.6%)	150 (22.9%)		

*—statistical significance *p* < 0.05; y.o.—years old.

**Table 3:** Frequency distribution of genotypes and alleles *GRIN2B* polymorphisms in patients with schizophrenia with early or adult age of onset of schizophrenia.

SNPs	Genotypes and Alleles	Early Onset (<18 y.o.)	Adult Onset (≥18 y.o.)	χ^2^	*p*-Value
rs7313149	AA	49 (71.0%)	192 (60.0%)	4.705	0.095
	AG	20 (29.0%)	115 (35.9%)		
	GG	0 (0%)	13 (4.1%)		
	A	118 (85.5%)	499 (78.0%)	3.931	0.048 *
	G	20 (14.5%)	141 (22.0%)		
rs2192970	AA	0 (0%)	7 (2.2%)	1.537	0.464
	AG	21 (30.4%)	94 (29.2%)		
	GG	48 (69.6%)	221 (68.6%)		
	A	21 (15.2%)	108 (16.8%)	0.066	0.798
	G	117 (84.8%)	563 (83.2%)		
rs10845838	AA	5 (7.1%)	40 (12.3%)	1.832	0.400
	AG	32 (45.7%)	151 (46.5%)		
	GG	33 (47.1%)	134 (41.2%)		
	A	42 (30.0%)	231 (35.5%)	1.562	0.212
	G	98 (70.0%)	419 (64.5%)		
rs10772715	AA	7 (10.0%)	60 (18.3%)	3.089	0.213
	AG	36 (51.4%)	161 (49.2%)		
	GG	27 (38.6%)	106 (32.4%)		
	A	50 (35.7%)	281 (43.0%)	2.495	0.115
	G	90 (64.3%)	373 (57.0%)		
rs1805481	AA	21 (30.9%)	86 (27.0%)	1.436	0.488
	AC	38 (55.9%)	171 (53.8%)		
	CC	9 (13.2%)	61 (19.2%)		
	A	80 (58.8%)	343 (53.9%)	1.083	0.299
	C	56 (41.2%)	293 (46.1%)		

*—statistical significance *p* < 0.05.

**Table 4:** Odds ratios calculated for *GRIN2A* and *GRIN2B* polymorphisms associated with early onset of schizophrenia.

Gene	SNPs	Genotypes and Alleles	OR	Cl (95%)	χ^2^	*p*-Value
*GRIN2A*	rs7206256	AA	1.573	0.929–2.663	6.279	0.043
		AG	0.995	0.592–1.673		
		GG	0.312	0.109–0.893		
		A	1.599	1.078–2.372	5.497	0.020
		G	0.625	0.422–0.928		
*GRIN2A*	rs11644461	AA	0.715	0.419–1.218	6.760	0.034
		AG	0.872	0.519–1.464		
		GG	2.312	1.203–4.443		
		A	0.664	0.460–0.960	4.767	0.030
		G	1.505	1.041–2.176		
*GRIN2B*	rs7313149	AA	1.633	0.927–2.877	4.705	0.095
		AG	0.728	0.412–1.284		
		GG	-	-		
		A	1.667	1.002–2.775	3.931	0.048
		G	0.600	0.360–0.998		

## Data Availability

The datasets generated for this study will not be made publicly available, but they are available on reasonable request to Svetlana A. Ivanova (ivanovaniipz@gmail.com), following approval of the Board of Directors of the MHRI, in line with local guidelines and regulations.
